# Supplementary material for: Label-free nanoscale mapping of intracellular organelle chemistry
Source: Commun Biol. 2023 May 31;6:583. doi: 10.1038/s42003-023-04943-7 (PMC10232547; doi:10.1038/s42003-023-04943-7)
Supplement: Supplementary file 2 — Supplementary Information [file 42003_2023_4943_MOESM2_ESM.pdf]

Supplementary Information

Label-free nanoscale mapping of intracellular organelle chemistry

George E. Greaves[1]*, Darya Kiryushko[1,2], Holger W. Auner[3], Alexandra E. Porter[2] and Chris C. Phillips[1]*

[1]Experimental Solid State Group, Department of Physics, Imperial College London, London, United Kingdom

[2]Department of Materials and London Centre for Nanotechnology, Imperial College London, London, United Kingdom

[3]Department of Immunology and Inflammation, The Hugh and Josseline Langmuir Centre for Myeloma Research, Imperial College London, London, United Kingdom

*Corresponding Authors: george.greaves15@imperial.ac.uk, chris.phillips@imperial.ac.uk

Supplementary Note 1: Section thickness

The thickness of multiple myeloma cell sections used for this study ranged between (70–200)nm. A range of thicknesses was desirable in the early stages of the project whilst we optimised the technique. We didn’t find that section thickness impacted image quality noticeably, however. To compare images of different cells, a single section thickness should be used.

Supplementary Note 2: The effect of a reflective substrate

The s-SNOM signal is highly surface sensitive. The contribution to the s-SNOM signal of a volume element in the sample decays rapidly with depth into the sample. For this reason, it is unlikely that using a reflective substrate improves our signal through a near-field interaction between the tip and the substrate. Instead, we expect that the dominant mechanisms for the improved signal are: (1) the increased illumination of the tip due to reflection from the substrate and (2) the reflection from the substrate of the backscattered light[1].

Supplementary Figure 1

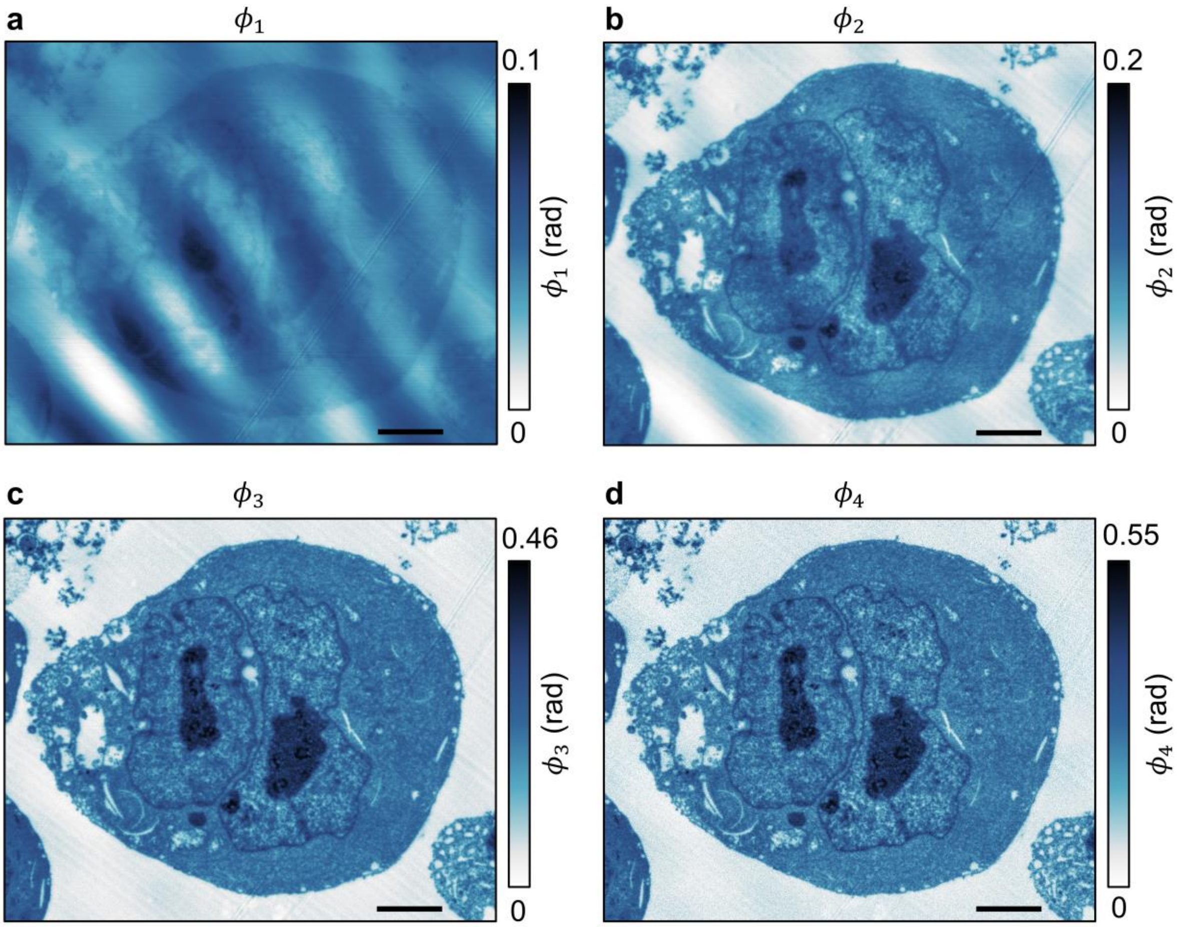

Supplementary Fig 1. s-SNOM images acquired at a wavelength of 1667cm^{-1} at each available harmonic of the probe oscillation frequency. At the first and second harmonics ((a) and (b) respectively), the background signal is not sufficiently removed by the pseudoheterodyne detection system. The background was deemed sufficiently removed at the third harmonic (c) and is presented in this study ahead of fourth harmonic images (d) due to a superior image signal-to-noise ratio. Scale bars 3µm.

Supplementary Figure 2

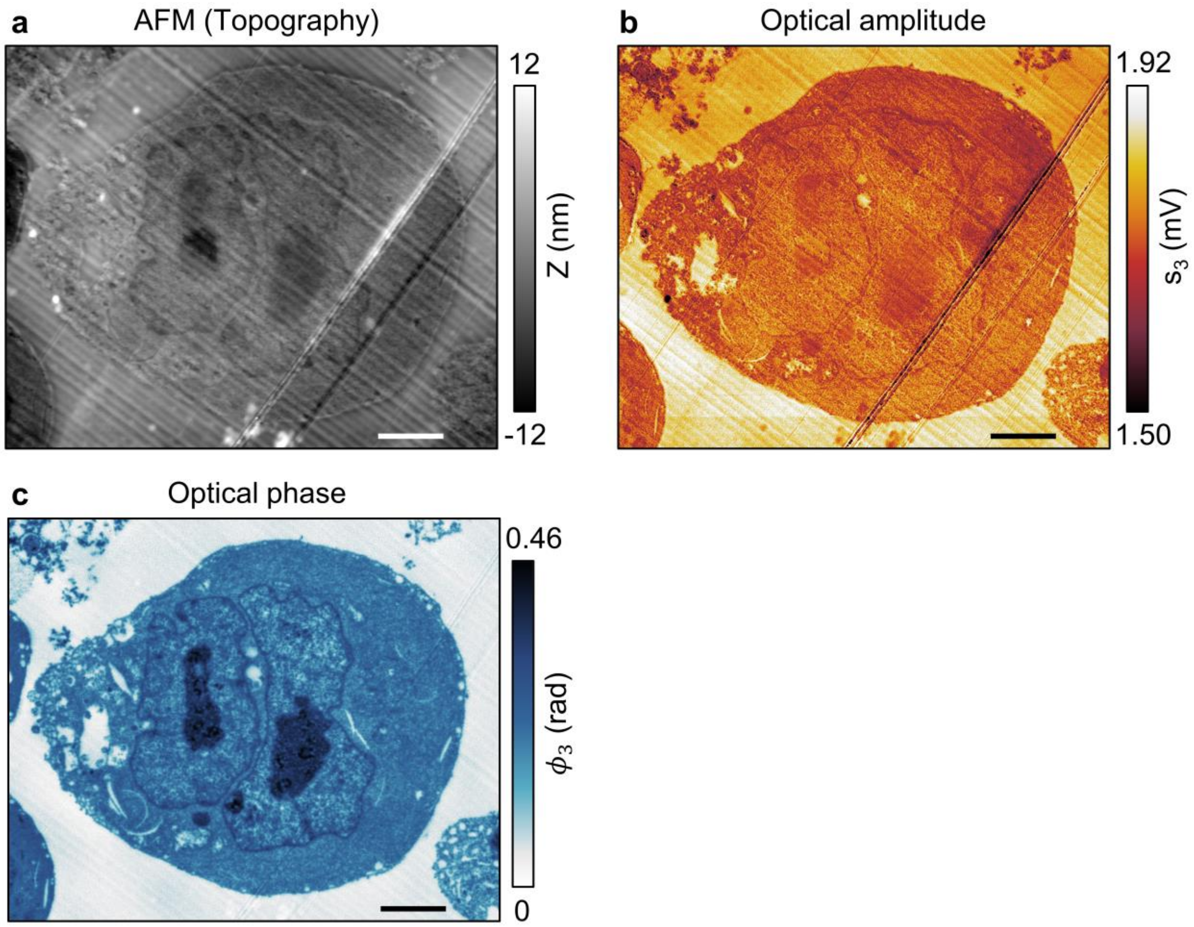

Supplementary Fig 2. A full set of topography, optical amplitude and optical phase images acquired at a wavelength of 1667cm^{-1}. The AFM image (a) shows the topography of the section. Some structures can be identified based on morphology which we expect is due to different shrinkage factors once the section has been cut, but there is a lack of chemical information. The images (b) and (c) are maps of the optical amplitude and optical phase respectively. In the case of weak oscillators such as the vibrational modes probed here, the optical amplitude and optical phase relate to wavelength in a way that is similar to the real and imaginary parts of the dielectric function of the sample respectively[2]. Since the imaginary part of the dielectric function exhibits a peak centred at the resonant frequency of the oscillator(s), tuning the imaging wavelength to the resonant frequency results in chemical contrast being displayed in the optical phase image. The real part of the dielectric function does not exhibit peaks at resonant frequencies, and it is thus more complicated to extract chemical information from this quantity. Scale bars 3µm.

Supplementary Figure 3

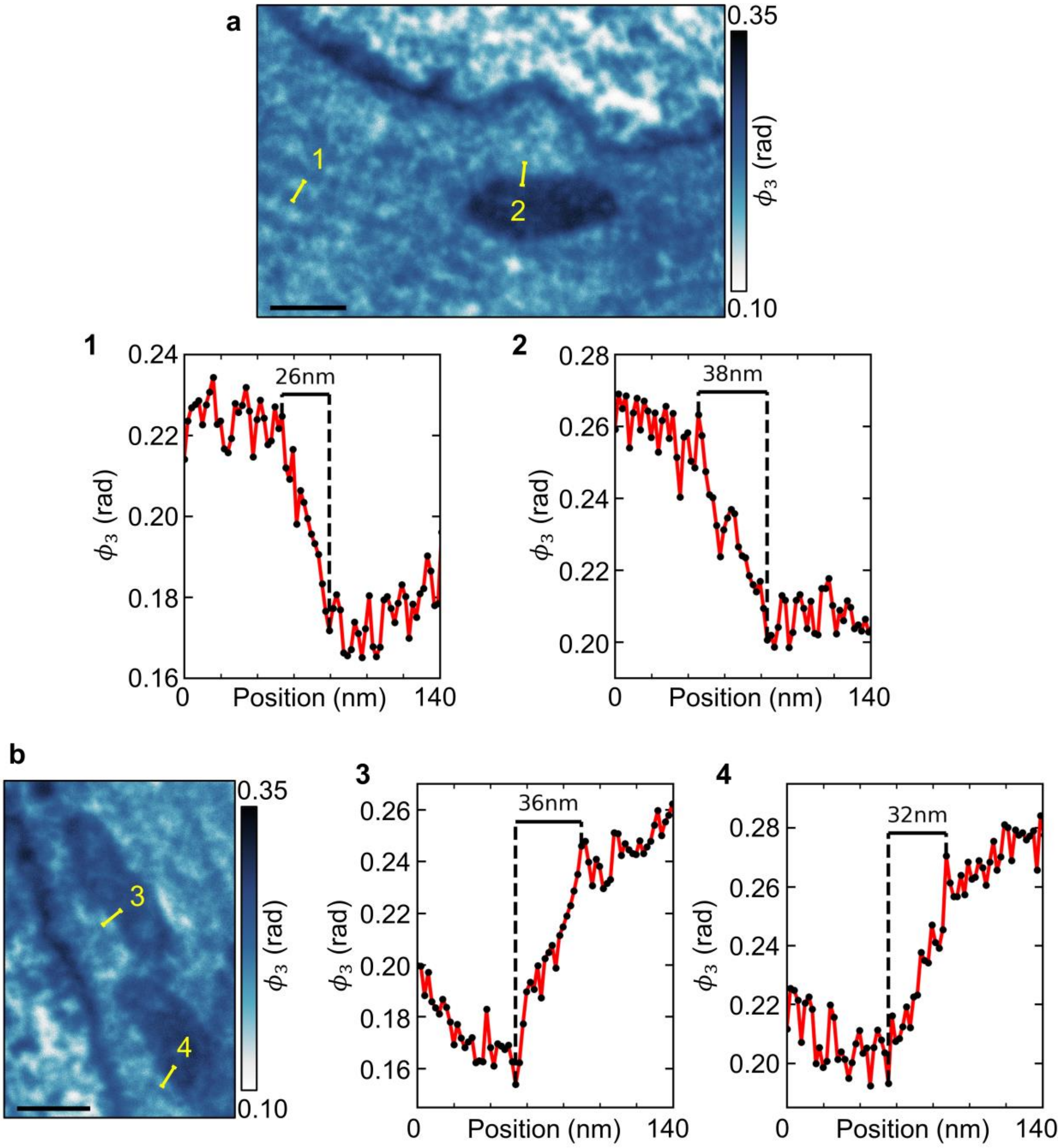

Supplementary Fig 3. Spatial profiles (1-4) of the s-SNOM signal across structures in a myeloma cell. Images (a,b) are acquired at 1667cm^{-1} to target amide groups. Profiles are averaged over a width of 6nm (3 pixels). Scale bars 500nm.

Supplementary Figure 4

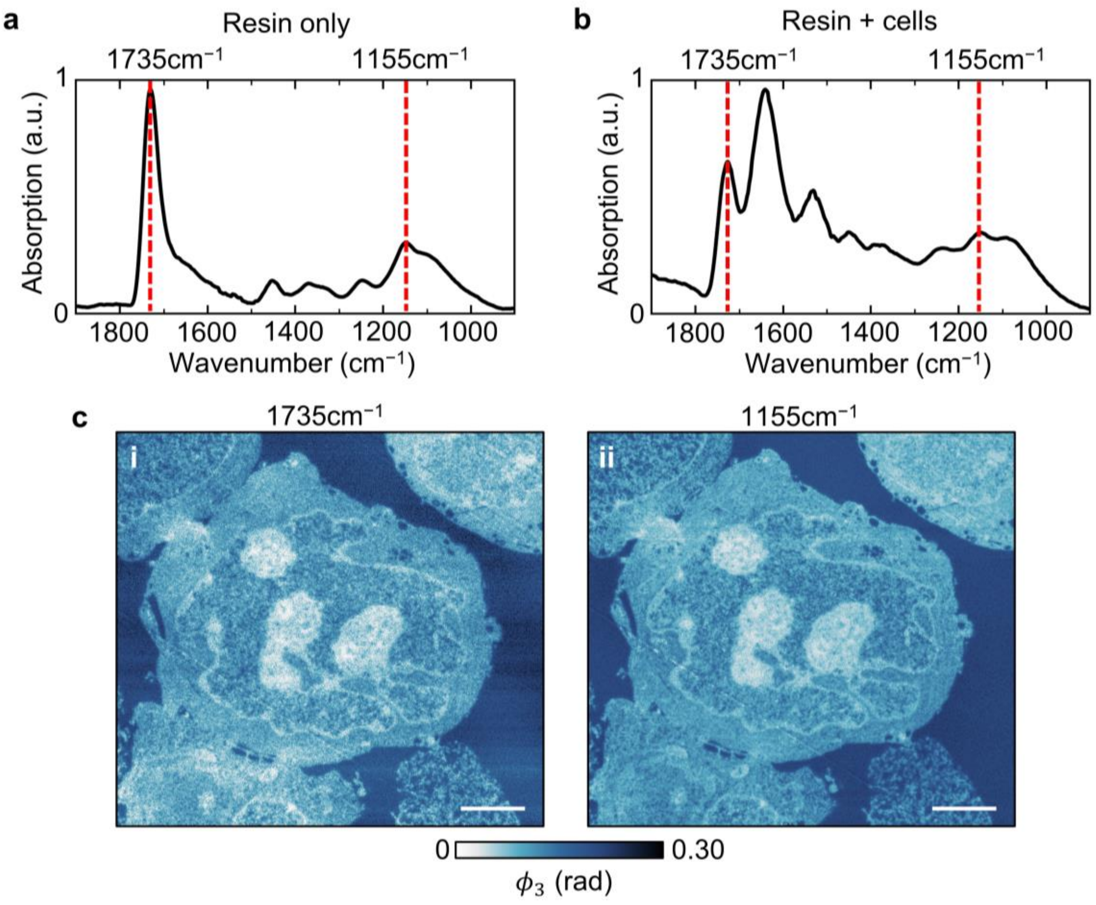

Supplementary Fig 4. Far-field absorption spectra of resin-embedded myeloma cell sections acquired with a Fourier transform infrared (FTIR) spectrometer (Vertex 70, Bruker, USA) with Hyperion IR microscope attachement, operated in reflection mode. Sections were cut to a thickness of 500nm using a Leica UC7 ultramicrotome (Leica, Austria) with a 35° diamond knife (Diatome, Switzerland) and collected on a gold-coated coverslip. The absorption spectra in (a) and (b) were acquired for regions of the section without and with cells respectively. We attribute the absorption band centred at 1735cm^{-1} to C=O vibrational modes in anhydride and aldehyde groups in the resin and fixatives respectively, and the band centred at 1155cm^{-1} to C–O–C stretching of epoxy groups in the resin. s-SNOM chemical mapping at 1735cm^{-1} (c(i)) and 1155cm^{-1} (c(ii)) shows the distribution of the embedding resin throughout the cell. Scale bars 2µm.

Supplementary Figure 5

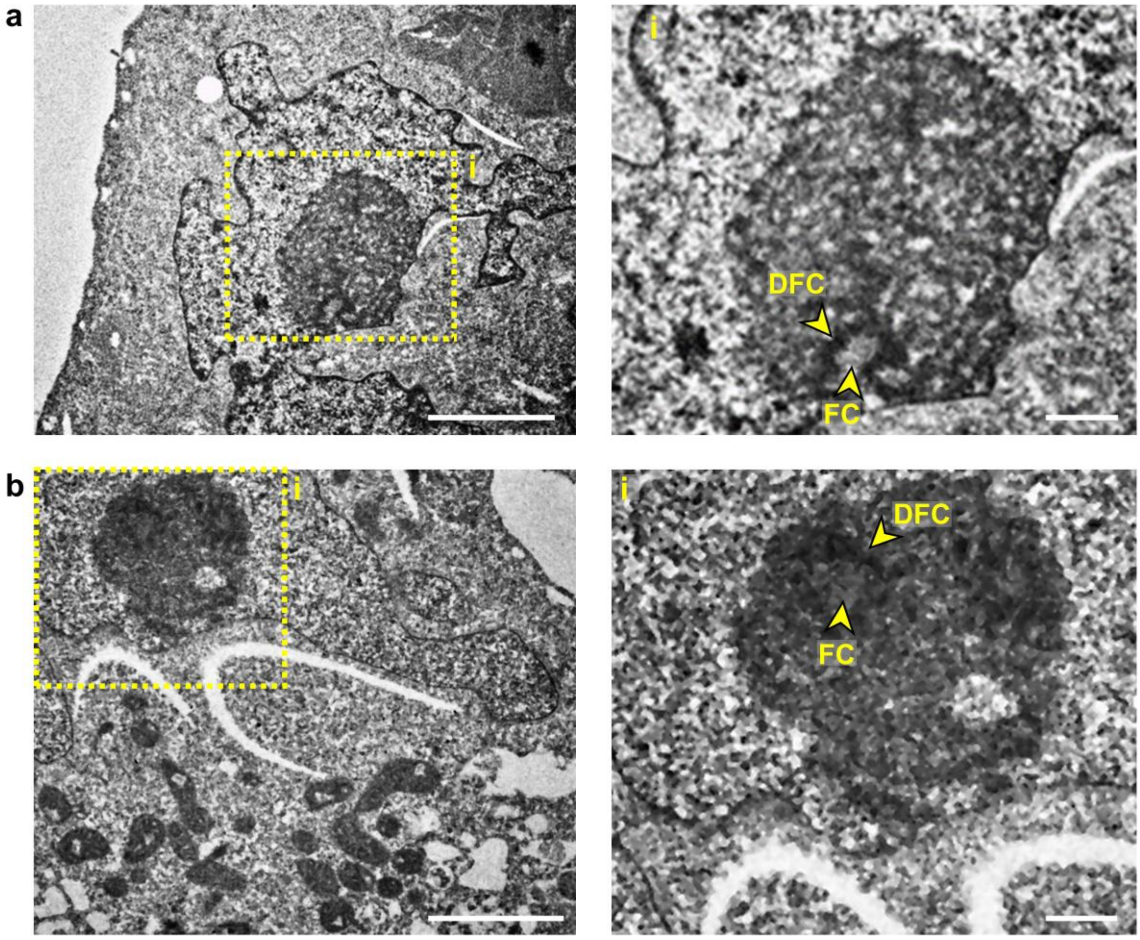

Supplementary Fig 5. TEM images (a,b) of myeloma cells poststained with uranyl acetate and lead citrate but left non-osmicated. Insets a(i) and b(i) show nucleoli in more detail. DFC; dense fibrillar component, FC; fibrillar centre. Scale bars 2µm (a,b) and 500nm (a(i), b(i)).

Supplementary Figure 6

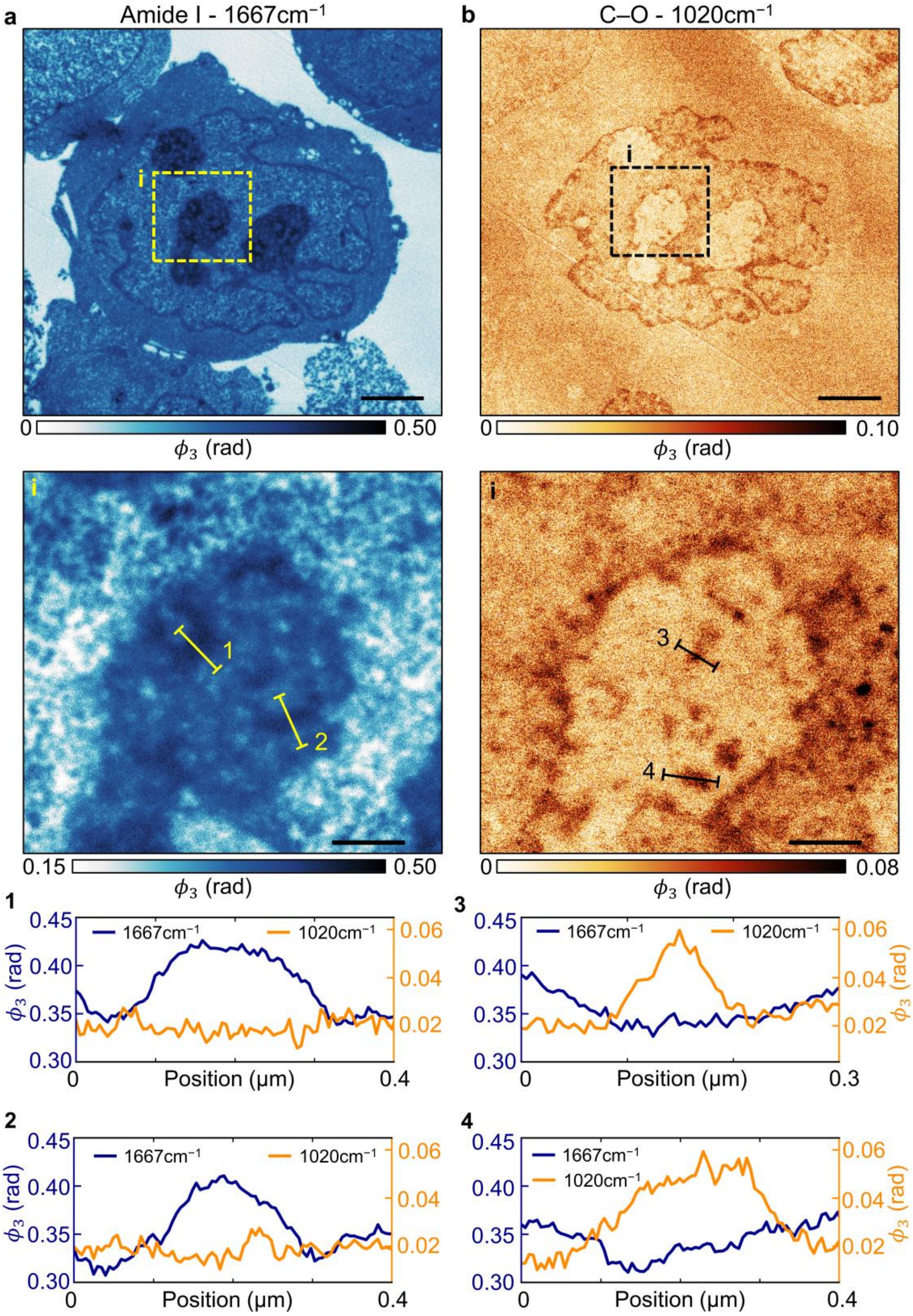

Supplementary Fig 6. Chemical mapping of a myeloma cell acquired at wavelengths exciting amide moieties (a) and C–O bonds (b). Insets a(i) and b(i) show structures in more detail. Spatial profiles (1-4) show the s-SNOM signal across structures at both imaging wavelengths. Profiles are averaged over a width of 75nm (15 pixels). Scale bars 2µm (a,b) and 500nm (a(i), b(i)).

Supplementary Figure 7

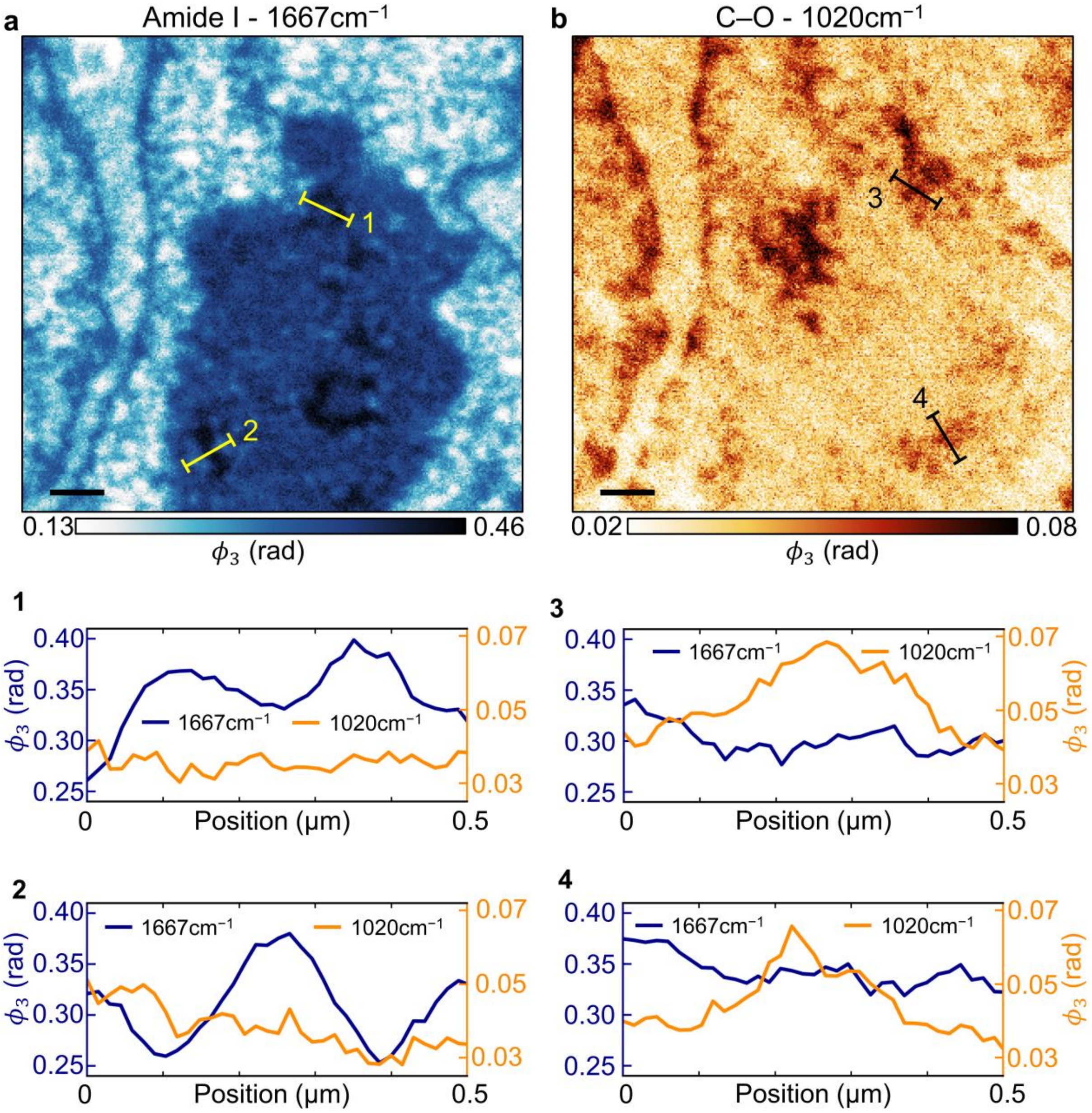

Supplementary Fig 7. Chemical mapping of a myeloma cell nucleolus acquired at wavelengths exciting amide moieties (a) and C–O bonds (b). Spatial profiles (1-4) show the s-SNOM signal across structures at both imaging wavelengths. Profiles are averaged over a width of 75nm (5 pixels). Scale bars 500nm.

Supplementary Figure 8

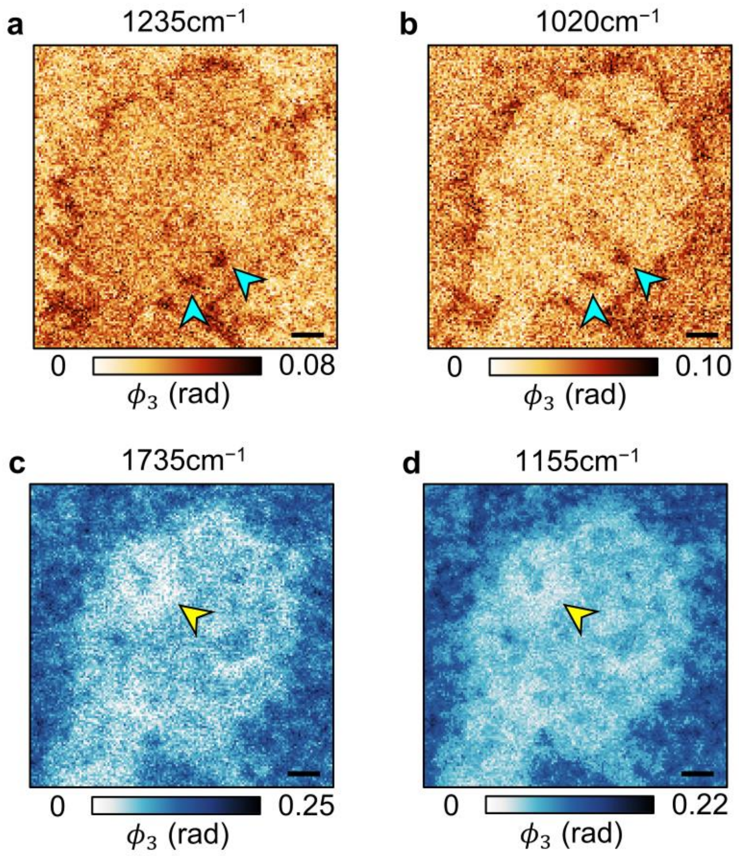

Supplementary Fig 8. Chemical mapping of a myeloma cell nucleolus at imaging wavelengths exciting phosphodiester moieties (a), C–O bonds (b), C=O bonds in the embedding resin and fixatives (c), and C–O–C bonds in the embedding resin (d). Whilst it is likely that the resin makes a small contribution to the s-SNOM signal at the imaging wavelengths in (a) and (b), this contribution doesn't appear to introduce artefacts as features in (c) and (d) indicated with yellow arrows are not present in (a) and (b). The structures indicated by the cyan arrows in (a) and (b) appear to be chemically specific as they are not identifiable in (c) and (d). Scale bars 250nm.

Supplementary References

1. Aizpurua, J., Taubner, T., García de Abajo, F. J., Brehm, M. & Hillenbrand, R. Substrate-enhanced infrared near-field spectroscopy. *Optics Express* **16**, 1529 (2008). https://doi.org:10.1364/OE.16.001529
2. Mester, L., Govyadinov, A. A. & Hillenbrand, R. High-fidelity nano-FTIR spectroscopy by on-pixel normalization of signal harmonics. *Nanophotonics* **11**, 377-390 (2021).
